# Mining the bacterial genome to discover new antimicrobial molecules

**DOI:** 10.15252/emmm.202115409

**Published:** 2021-12-15

**Authors:** Wenjie Liang, Julien Diana

**Affiliations:** ^1^ Institut Necker Enfants Malades (INEM) Institut National de la Santé et de la Recherche Médicale (INSERM) Centre National de la Recherche Scientifique (CNRS) Université de Paris Paris France

**Keywords:** Microbiology, Virology & Host Pathogen Interaction

## Abstract

Multidrug resistance is one of the major public health issues the world is facing today. However, the World Health Organization (WHO) revealed recently that there has been little progress in the development of new antibiotics to tackle drug‐resistant infections. By mining the bacterial genome database, Zhu *et al*, in the last issue of *EMBO Molecular Medicine*, report a defensin expressed by human oral actinomyces, actinomycesin, and characterize its anti‐infectious capacity (Zhu *et al*, 2021). They demonstrate the safety and efficacy of this bacterial antimicrobial peptide (AMP) against various bacterial strains, describe its mode of action, and validate its use as systemic drug therapy against bacterial infections in mice. This study highlights human oral bacteria as a source of antimicrobial agents that need to be considered in the future to fight multidrug‐resistant bacteria.

## Graphical abstract

W. Liang and J. Diana discuss the study by Zhu *et al* (in this issue of *EMBO Mol Med*) that highlights human oral bacteria as a powerful source of antimicrobial agents to fight multidrug‐resistant bacteria.

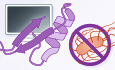

Antimicrobial peptide (AMPs), also known as host defense peptides, are attractive therapeutic candidates thanks to their potent broad‐spectrum activity against bacteria, fungi, protozoa, parasites, and viruses (Zasloff, 2006). The ability of AMPs to kill bacteria relies on their capacity to bind and penetrate the bacterial membrane or cell wall, and to subsequently alter membrane formation, and protein biosynthesis process. AMPs are cationic and amphipathic molecules and are typically from 10 to 100 amino acids in length, and are evolutionarily conserved from microorganisms, plants, and invertebrates to more complex amphibians and mammals. Beyond their microbicidal function, AMPs exhibit complex immunomodulatory functions in vertebrates on both innate and adaptive immunity, including, for example, adjuvant activity or recruitment of immune cells (Hancock *et al*, 2016). The direct microbicidal action of vertebrate AMPs is weak under physiological conditions, as their positive charge is modified, and their bactericidal capacities are buffered in the presence of host factors. Consequently, their immunomodulatory effect is critical to build an efficient anti‐infective response. The immunomodulatory facet of eukaryotic AMPs and their poor microbicidal activity under physiological conditions limit their attractivity as safe and efficient systemically administered antibiotics. Consistently, after decades of research, most vertebrate AMPs tested in clinical trials have been formulated for topical applications in the treatment of lung, oral, or skin infections (Mookherjee *et al*, 2020).

In this issue of *EMBO Molecular Medicine*, Zhu *et al* propose the use of human oral‐derived bacterial AMPs as safe and efficient antibiotics to treat multidrug‐resistant infections (Zhu *et al*, 2021) (Fig 1). Contrary to their vertebrate counterparts that evolved as immunomodulatory molecules, bacterial AMPs have naturally evolved to optimize their antimicrobial capacity providing an efficient competitive advantage toward their producer’s competitors. Therefore, the authors conducted a systematic mining of the microbial genome database using the vertebrate AMPs defensins as queries. They identified microbial defensins in the actinobacteria and myxobacteria subgroups, most of them being derived from actinomyces colonized in human oral cavity. Using mathematical models of codon substitution, they further demonstrated that unlike vertebrate defensins, bacterial defensins evolved under positive Darwinian selection to optimize their antibiotic activity to adapt to the complex oral multispecies communities. Bacterial defensins from oral actinomycetes could, therefore, carry a higher antibiotic potential than eukaryotic defensins, as their antibacterial function has been improved by microflora‐driven natural selection. Among 48 bacterial defensins, the authors selected a representative member for further functional characterization, actinomycesin.

**Figure 1:**
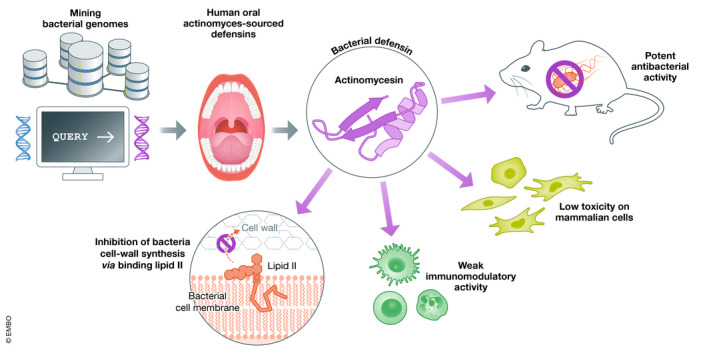
Therapeutic potential of the human oral sourced defensins as systemic antibiotic Mining of bacteria genome database led to the discovery of actinomycesin, a human oral‐derived actinomyces‐sourced defensin. This bacterial antimicrobial peptide (AMP) shows potent antibacterial activity both *in vitro* and *in vivo* via the inhibition of bacteria cell wall synthesis. Actinomycesin shows low toxicity against human cell lines and weak immunomodulatory activity, supporting the use of actinomycesin and potentially other bacteria AMPs as systemic antibiotics against multidrug‐resistant bacteria.

First, using a classical inhibition‐zone assay, the authors demonstrated *in vitro* the antibacterial potency of actinomycesin against various bacteria strains clinically relevant in human infections. Actinomycesin exhibited high activity against *Streptococcus* and *Staphylococcus* strains, including antibiotic‐resistant strains, whereas at similar concentrations, oral human AMPs such as defensins HNP1&2 or lysozyme C were not efficient. The authors, however, nicely demonstrated an antibacterial synergy between bacterial and human AMPs that was not due to physical interaction as demonstrated by bio‐affinity electrospray ionization mass spectrometry. They conducted an elegant series of experiments to demonstrate that the bactericidal activity of actinomycesin is mediated by the inhibition of bacterial cell wall synthesis, likely via the binding of the precursor molecule membrane‐anchored Lipid II. Importantly, this mechanism of action may explain the synergistic effect between actinomycesin and human AMPs. Indeed, lysozyme may facilitate actinomycesin access to Lipid II by partial hydrolysis of bacterial cell wall peptidoglycan, and HNP1&2 could facilitate the binding of actinomycesin by changing the conformation of Lipid II. Additional studies are required to confirm this stimulating hypothesis. Importantly, the authors show that, contrary to vertebrate AMPs, host molecules present in serum or pure DNA did not alter the microbicidal capacity of actinomycesin.

Since actinomycesin targets bacterial cell wall synthesis and does not disrupt bacterial membrane as many other AMPs, it could reduce the risk of toxicity on eukaryotic cells. The authors confirmed the low toxicity of actinomycesin on human kidney cell line and demonstrated that actinomycesin had no effect on ion channels expressed in human central nervous system and heart.

As mentioned above, the direct bactericidal activity of eucaryotic AMPs is reduced in physiological conditions. However, they exhibit either pro‐inflammatory or anti‐inflammatory activities that promote an efficient and controlled anti‐infectious immune response. Eucaryotic AMPs control the recruitment of various immune cell types at the site of infection, modulate the activity of neutrophils, enhance the antigen presentation ability of dendritic cells, but also induce regulatory mechanisms that terminate the immune response. While these properties are beneficial in infectious context, aberrant expression of vertebrate AMPs in sterile condition may be detrimental as shown in various autoimmune contexts (Liang & Diana, 2020) and this impedes their use as systemic drugs. Due to the different environment of natural evolution, bacterial AMPs may show lower immunomodulatory abilities. Zhu *et al* evaluated the release of IL‐8 by human alveolar type II cell line treated by actinomycesin or by the human AMP LL‐37. They observed that even at high dose, actinomycesin induced only low level of IL‐8 compared with LL‐37. Using surface plasmon resonance analysis and bioinformatic prediction, the authors further showed that actinomycesin exhibited low immunogenicity and low allergenicity. However, follow‐up studies, including *in vivo* experiments, will be required to confirm the minimal impact of actinomycesin on the complex immune system of vertebrates.

Finally, the antibiotic potential of actinomycesin was evaluated in preclinical mouse models of *Streptococcus pneumoniae*‐induced pneumonia and methicillin‐resistant *Staphylococcus aureus*‐induced peritonitis. In both infectious models, actinomycesin treatment showed high therapeutic efficacy, reducing bacterial load and improving mouse survival. Future studies will be required to evaluate the potential use of actinomycesin against other pathogenic bacteria and pathogens. As already performed with vertebrate AMPs, the sequences, and structures of bacterial defensins may allow the development of novel therapeutic synthetic peptides with optimized bactericidal capacity and reduced immunogenicity (Haney *et al*, 2019).

In conclusion, the study of Zhu *et al*. provides a proof of concept that mining the microbial genome is a promising approach to discover efficient AMPs for systemic therapy of bacterial infectious diseases.
